# Management of Physiological Hyperpigmentation of Oral Mucosa by Cryosurgical Treatment: A Case Report

**DOI:** 10.5681/joddd.2012.030

**Published:** 2012-11-12

**Authors:** Maryam Talebi, Niloofar Farmanbar, Salman Abolfazli, Alireza Sarraf Shirazi

**Affiliations:** ^1^Associate Professor, Department of Pediatric Dentistry, Faculty of Dentistry, Mashhad University of Medical Sciences, Mashhad, Iran; ^2^Post-graduate Student, Department of Pediatric Dentistry, Faculty of Dentistry, Mashhad University of Medical Sciences, Mashhad, Iran; ^3^Post-graduate Student, Department of Periodontics, Faculty of Dentistry, Mashhad University of Medical Sciences, Mashhad, Iran

**Keywords:** Cryosurgery, depigmentation, liquid nitrogen, gingiva

## Abstract

Melanin hyperpigmentation is the result of melanin granules. "Black gums" may cause esthetic problems. Different treatment modalities have been used with the aim of removing pigmentations for esthetic reasons, all of which have some advantages and disadvantages. Recurrent lesions are the most important concept in all of these treatments. Cryotherapy is a method of tissue destruction by rapid freezing. It is an atraumatic, cost-effective and simple method for treating oral pigmentation. This report presents the effects of cryotherapy on physiologic pigmentations of oral mucosa in a 9-year-old boy. In this case no recurrent lesions were observed after 12 months.

## Introduction

Melanin hyperpigmentation of gingiva usually does not present a medical problem, but many patients may considertheir black gums to be unaesthetic. Melanin pigmentation of gingiva is symmetric and persistent, and it does not alter normal gingival architecture.^1^

Various depigmentation techniques have been employed with similar results, such as gingivectomy, gingivectomy with free gingival autografting, electrosurgery, cryosurgery, chemical agents such as 90% phenol and 95% alcohol, abrasion with a diamond bur, Nd:Yag laser, semiconductor diode laser and CO_2_ laser.^2^

Cryotherapy is a method of tissue destruction by rapid freezing. The cytoplasm of the cell freezes, leading to denaturation of proteins and cell death. This procedure does not require use of local anesthesia, is relatively a painless procedure, and has been shown to produce excellent results lasting for several years. This procedure does not even require a periodontal dressing. However, the removal of pigments cannot be evaluated during the procedure and requires a separate sitting after about 5 days, during which the residual pigmentation should be removed.^3^ Cryotherapy has been introduced as an effective therapeutic method in treating oral mucosal lesions such as leukoplakia, pyogenic granuloma, and peripheral giant cell granuloma.^4^

This report describes the effects of cryotherapy on physiologic pigmentation of oral mucosa and it is hoped that the results will be used to suggest an effective method for treatment of similar oral mucosal lesions.

## Case report

**Figure 1:**
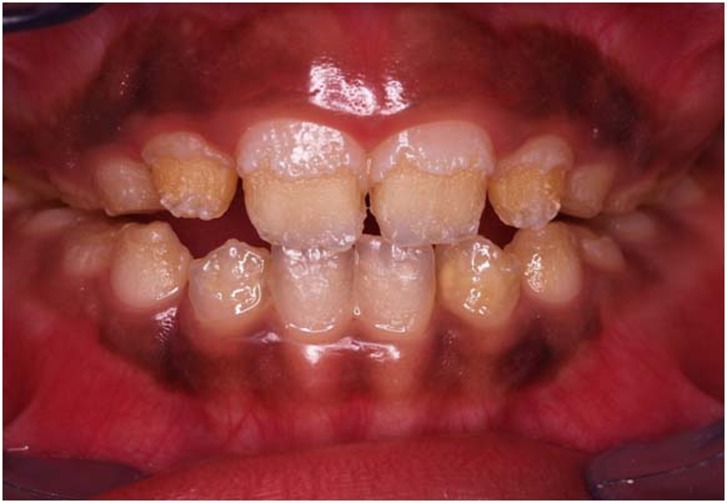
Gingival view before cryosurgical treatment.

A 9-year-old boy was referred to the Department of Pediatric Dentistry, Mashhad University of Medical Sciences, Mashhad, Iran. The patient needed comprehensive dental treatment due to amelogenesis imperfecta. At the same time his parents requested to treat his gingival pigmentation. His oral examination revealed that his gingiva is deeply pigmented from the right second primary molar to the left second primary molar (Figure 1). He had been checked by a physician previously and there were no pathologic disorders (e.g. Albright syndrome, trauma, hemachromatosis and racial pigmentation) in this case. Therefore, we started depigmentation therapy by cryosurgery technique.

Liquid nitrogen was provided in the morning of treatment day by a consumer gas factory and was transferred to the dental facility. In order to transfer the liquid nitrogen, it had been saved in a two-layer flask (metallic inner layer and glassy outer layer).

### Treatment process

**Figure 2:**
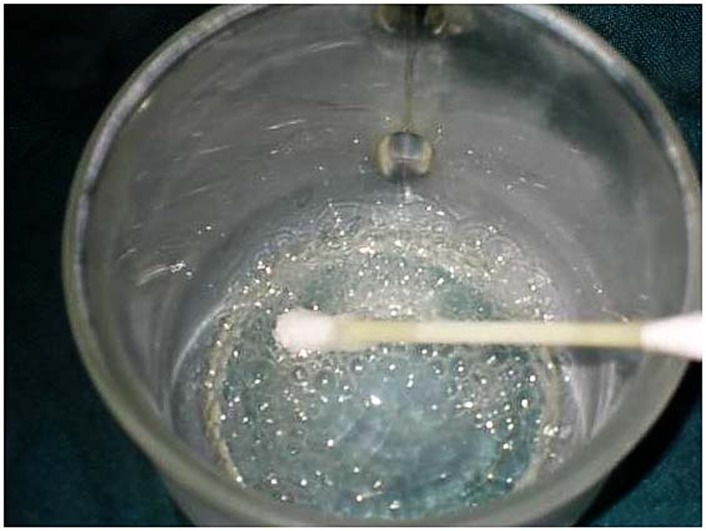
Liquid nitrogen was poured into the glassy mug.

**Figure 3:**
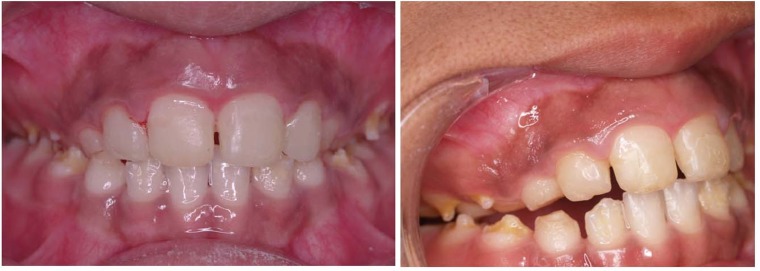
Gingival view after cryosurgical treatment.

In the first step isolation was instated by suction and cotton rolls; then hyperpigmentation areas were dried byair spray and 10% topical lidocaine gel was used to achieve anesthesia. During treatment, liquid nitrogen was poured into the glass mug and was rolled on the pigmentation area by cotton swabs (Figure 2). After the gingiva blanched, the operation was started in another area; after 20 seconds this process was repeated. It is possible repeat this process three or four times for each area. The total time needed for this operation was 30 minutes. In order to record and analyze the results, five standard photos were taken in different positions. Standard high-quality images were taken at baseline and after one, three, six and twelve months (Figure 3). All the photos were taken by a Canon digital camera (Ixus 800IS, Japan) in the same position, while the patient was sitting upright, and the distance between patient and camera was fixed to about 30 centimeters.

**Figure 4:**
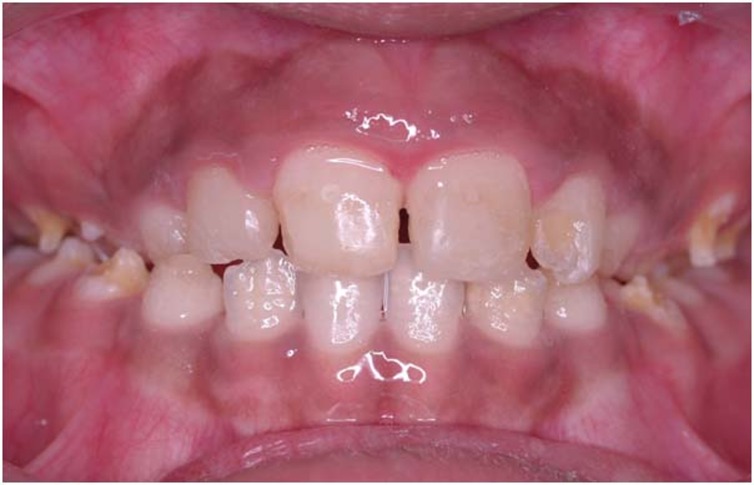
Oral pigmentation without recurrent lesions after 12 months.

Minimal reddish erythema was observed after cryosurgery. After one month the gingival color was ideal. No complications were observed during the follow-up. No recurrent lesions were observed in 12-month follow-up (Figure 4).

## Discussion

Melanin pigmentation is the result of melanin granules, produced by melanoblasts intertwined between epithelial cells at the basal layer of the epithelium. Complaints of "black gums" may cause esthetic problems and embarrassment, particularly if the pigmentations are visible during speech and smiling. Demand for cosmetic therapy of gingival melanin pigmentation is high. In this report we used cryosurgery by liquid nitrogen-cooled cotton swabs.

Liquid nitrogen has a low cost and does not need specialized tools and techniques, while laser and surgery options need additional accuracy and specialized tools. Although Mokeem believes laser and cryosurgical treatment modalities achieve satisfactory results, these methods require sophisticated equipment which is not commonly available in hospitals and clinics.^2^ Surgery is another selective option. It is obvious that surgery techniques, gingivectomy and diamond bur abrasion have aggressive natures, which can damage the bone underneath and cause keratinized tissue loss.^5^

Humagain reported that scalpel surgery causes unpleasant bleeding during and after the operation, and it is necessary to cover the surgical site with periodontal dressings for 7 to 10 days.^1^ Khalid Almas believes scalpel surgical technique is highly recommended considering equipment deficiencies in developing countries, reporting that it is simple and easy to perform, cost-effective, with minimum discomfort and esthetically acceptable to the patient, while cryosurgery may be followed by considerable swelling and it is also accompanied by increased soft tissue destruction, depth control is difficult and optimal duration of freezing is not known; however, prolonged freezing increases tissue destruction.^6^

Tal did not observe recurrent lesions after 20 and 30 months, and the same held in cryosurgery treatment.^7^ Sarraf et al had a case series study in which treatment of gingival pigmentation of adolescent patients was very satisfactory.^5^ A study by Darbandi and Shahbaz showed that after four weeks all the pigmentated areas were cured, with no recurrent lesions in any of the patients.^5^ In studies by Kavashima and Tal a slight recurrence was observed after 3 and 6 months, by Er:YAG laser treatment, respectively.^8,9^ Nakamura used CO_2_ laser and did not report any recurrent lesions 12 months after the treatment, but after 2 years, recurrent lesions were observed in 4 patients.^10^ Mokeem did not see any recurrent lesions 18 months after the treatment by bur abrasion technique,^2^ while Pontes reported completely recurrent lesions after 12 months in all the samples.^11^

These different treatment modalities have been used with the aim of removing pigmentations for esthetic reasons and all of them have some advantages and disadvantages. Recurrent lesions are the most important concerns in all these treatment modalities. In this case no recurrent lesions were observed after one year.

## Conclusion

This report described cryotherapy as an atraumatic, cost-effective and simple method for treating oral pigmentation without recurrent lesions after 12 months.
